# Refining Mechanism of 7075 Al Alloy by In-Situ TiB_2_ Particles

**DOI:** 10.3390/ma10020132

**Published:** 2017-02-04

**Authors:** Guisheng Gan, Bin Yang, Bo Zhang, Xin Jiang, Yunlong Shi, Yiping Wu

**Affiliations:** 1 Chongqing Municipal Engineering Research Center of Institutions of Higher Education for Special Welding Materials and Technology, Chongqing University of Technology, Chongqing 400054, China; baiguozhenke@163.com (B.Z.); jiangxin@163.com (X.J.); 18324133469@163.com (Y.S.); 2 College of Materials Science and Engineering, Huazhong University of Science and Technology, Wuhan 430074, China; ypwu@mail.hust.edu.cn; 3 Collaborative Innovation Center of Steel Technology, University of Science & Technology Beijing, Beijing 100083, China; byang@ustb.edu.cn

**Keywords:** TiB_2_ particles, composites, microstructure, nucleation undercooling

## Abstract

The nucleation undercooling of TiB_2_/7075 Al matrix composites, the microstructure observed after solidification at different cooling rate, and the size and distribution of TiB_2_ particles were investigated. The experimental results have shown that the grain sizes of TiB_2_/7075 Al matrix composites firstly decreased, then increased, and finally decreased again with the increase of TiB_2_ content. The nucleation undercooling of TiB_2_/7075 Al matrix composites first increased, then decreased, and finally increased again with the increase of TiB_2_ content when the cooling rates was 5 and 10 °C/min respectively, but kept decreasing with the increase of TiB_2_ content at a cooling rate of 20 °C/min. The melting and solidification process showed no significant change with the decrease of cooling rate in 9.0% TiB_2_/7075 Al matrix composites. Most small particles can act as heterogeneous nucleus, which induced grain growth and were captured into the grain by the solid/liquid interface. At the same time, most of the larger particles and a minority of the small TiB_2_ particles are pushed into the grain boundary; locating in the grain boundary can hinder the Al atoms from diffusing during the solidification process and restrain α-Al phase growth. The influence of particles shifted from dominating by locating to dominating by nucleation as the quantity of TiB_2_ particles increased.

## 1. Introduction

TiB_2_ particles can improve the strength and modulus of aluminum alloy due to high hardness and modulus, and have been widely used in aluminum alloy as reinforcement and grain refiner, especially in the aeronautical industry as structural materials [1,2,3,4,5]. The effects of reactive conditions such as reaction temperature and the proportion of reactants on the refining effect of TiB_2_ particles have been studied extensively [6,7,8,9,10,11,12]. However, until now, there has been no consensus on the exact mechanism for grain refinement involving the addition of Al-Ti-B-type grain refiners. Some calculations suggested that only less than one percent of TiB_2_ particles nucleated a grain [13], but it strongly depended on two conditions in order to obtain efficient grain refinement: that a sufficient number of potential nuclei must be present in the melt, and a large fraction of the potential nuclei must be activated and effective [14]. A free-growth model based on the assumption of isothermal melt has been presented to predict the grain size on grain refinement by Maxwell and Greer et al. [15,16,17]. Greer et al. investigated the effects of the hypothetical size distributions of TiB_2_ particles on as-cast grain size in Al alloys, and found that an appropriate mean particle size and a narrow spread of the size distribution are preferable for an excellent grain refiner [18,19,20]. The particle density and the nucleation undercooling of the refining particles were two main parameters to control the transition from dendrites to equiaxed growth [21,22,23]. However, the density of effective particles cannot be easily estimated. In this paper, we present the nucleation undercooling of TiB_2_/7075 Al matrix composites by using differential scanning calorimetry (DSC), compare to the microstructure after solidification, and then investigate the size and distribution of TiB_2_ particles. Finally, the refining mechanism of the composites is discussed.

## 2. Experimental

K_2_TiF_6_ (mass > 97%) and KBF_4_ (mass > 97%) mixture were put into molten 7075 Al alloy with the composition Al-5.52Zn-2.36Mg-1.51Cu-0.18Si-0.26Fe-0.15Mn-0.25Cr (in mass %) at 850 °C. After being stirred at the same temperature for 30 min and degassed by using C_2_Cl_6_, 3.0% (in mass, similarly hereafter) and 4.5%, 6.0% and 9.0% TiB_2_/7075 Al matrix composites were prepared. The molten TiB_2_/7075 Al composites at 720 °C were casted in a graphite mold 15 mm in diameter. The liquidus and solidus temperatures of composites were measured by NETZSCH Q100 differential scanning calorimetry (DSC) with weight of about 8–9 mg, and the heating and cooling rates were 1 and 5, 10 and 20 °C/min between 400 °C and 700 °C, respectively. Samples were held for 3 min under the protection of argon when the temperature reached 400 °C with the same heating rate, then all samples were heated to 700 °C with the different heating rate (1 and 5, 10 and 20 °C/min, respectively). Finally, the temperature was decreased to 400 °C with the different cooling rate (1 and 5, 10 and 20 °C/min, respectively) after holding for 3 min again. After sanding and polishing, the specimens were etched in a solution of mixed acids (1% HF + 1.5% HCl + 2.5% HNO_3_ + 95% H_2_O). The microstructures of composites were tested by ZEISS SUPRA55 scanning electron microscope (SEM) and CIKONG 4XCE optical microscope (OM). The grain size and area of the primary solid phase were analyzed statistically by a quantitative image analysis system (Image-Pro plus 6.0). The mean grain size was denoted by the average gain diameter $D=2\sqrt{\frac{S}{\pi }}$, where *S* is the area of the primary phase.

## 3. Results and Discussion

### 3.1. The Microstructures of TiB_2_/7075 Al Matrix Composites with Different Cooling Rate

**Figure 1:**
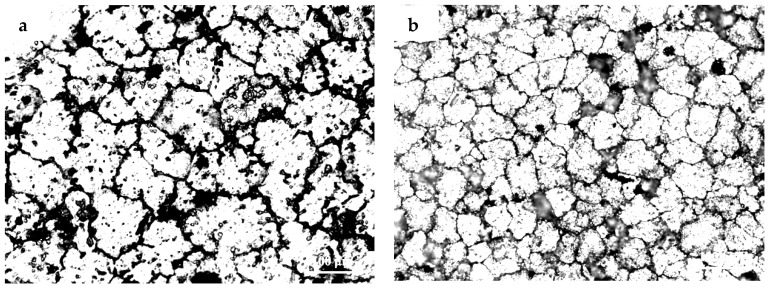

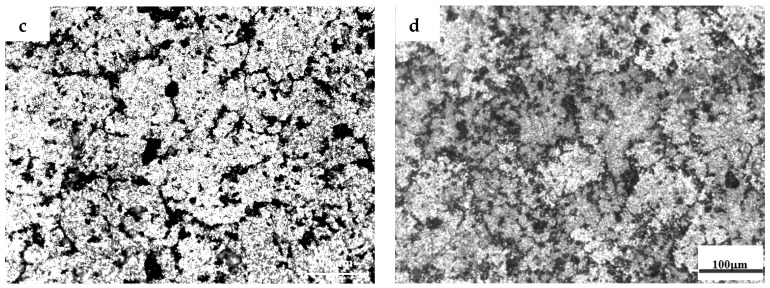
The microstructures of TiB_2_/7075 Al matrix composites. (**a**) 3.0% TiB_2_/7075; (**b**) 4.5% TiB_2_/7075; (**c**) 6.0% TiB_2_/7075; (**d**) 9.0% TiB_2_/7075.

The microstructures of 3.0% and 4.5%, 6.0% and 9.0% TiB_2_/7075 Al matrix composites after being poured into the graphite mold are pictured in Figure 1. The average grain sizes of 3.0% and 4.5% TiB_2_/7075 Al matrix composites were 69 μm and 50 μm, respectively. However, larger dendrite grains could be found after adding 6.0% TiB_2_; the 9.0% TiB_2_/7075 Al matrix composite was also mainly composed of the dendrite grains smaller than that of 6.0% TiB_2_/7075 Al matrix composites.

**Figure 2:**
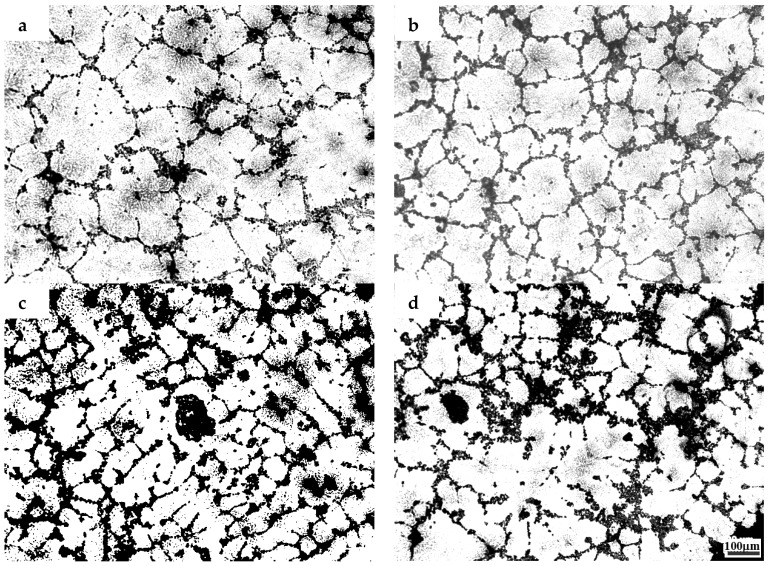
The microstructures of TiB_2_/7075 Al matrix composites with cooling rate of 20 °C/min. (**a**) 3.0% TiB_2_/7075; (**b**) 4.5% TiB_2_/7075; (**c**) 6.0% TiB_2_/7075; (**d**) 9.0% TiB_2_/7075.

**Figure 3:**
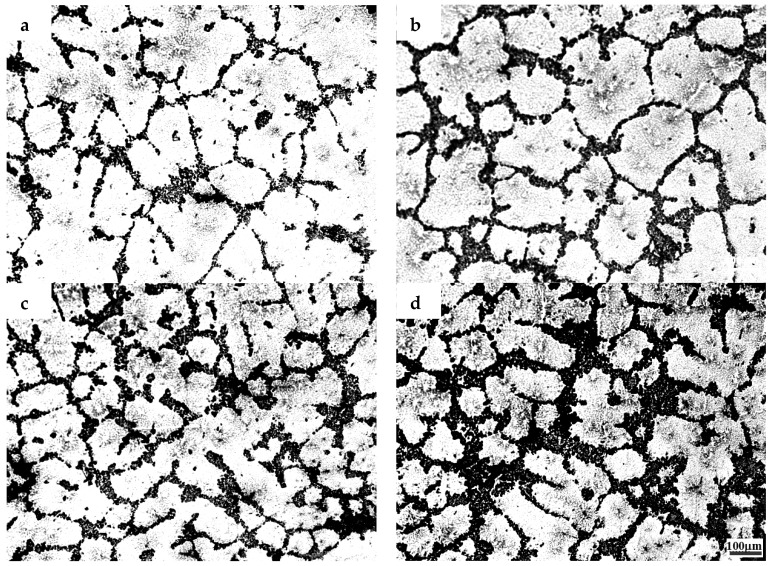
The microstructures of TiB_2_/7075 composites with cooling rate of 5 °C/min. (**a**) 3.0% TiB_2_/7075; (**b**) 4.5% TiB_2_/7075; (**c**) 6.0% TiB_2_/7075; (**d**) 9.0% TiB_2_/7075.

**Figure 4:**
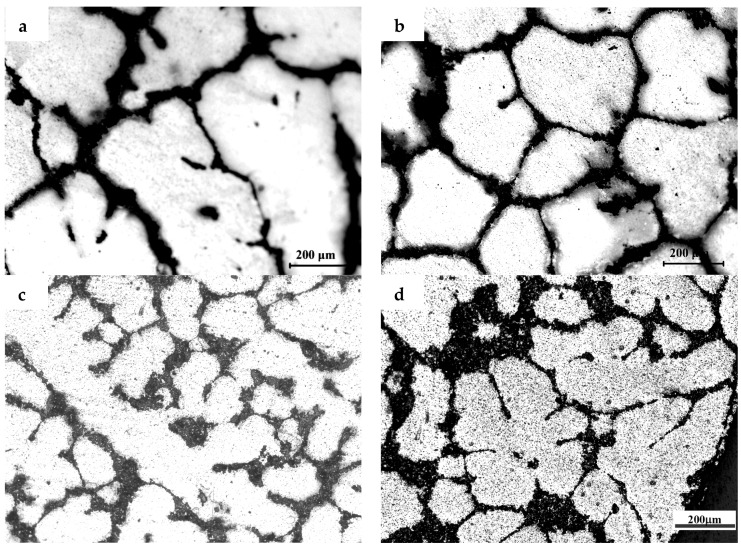
The microstructures of TiB_2_/7075 composites with cooling rate of 1 °C/min. (**a**) 3.0% TiB_2_/7075; (**b**) 4.5% TiB_2_/7075; (**c**) 6.0% TiB_2_/7075; (**d**) 9.0% TiB_2_/7075.

The microstructures of the samples after their solidification inside the DSC crucible have been compared. Figure 2, Figure 3 and Figure 4 show the microstructures of TiB_2_/7075 Al matrix composites with cooling rate of 20 °C/min, 5 °C/min and 1 °C/min from 700 °C to 400 °C respectively. The grain sizes of TiB_2_/7075 Al matrix composites firstly decrease, then increase, and finally decrease with the increase of TiB_2_ content. The mean grain sizes of 3.0% and 4.5% TiB_2_/7075 Al matrix composites reached 107 μm and 66 μm, respectively, with a cooling rate of 20 °C/min in Figure 2. The grain sizes of TiB_2_/7075 Al matrix composites increased with the decrease of cooling rate in Figure 3 and Figure 4, and the mean grain sizes of 3.0% and 4.5% TiB_2_/7075 Al matrix composites reached 236 μm and 165 μm, respectively, with cooling rate of 1 °C/min in Figure 4. Large dendrites can be found in 6.0% TiB_2_/7075 Al matrix composites. Rosette grains in 9.0% TiB_2_/7075 Al matrix composites are also very strongly apparent in Figure 4.

### 3.2. The Undercooling of TiB_2_/7075 Al Matrix Composites with Different Cooling Rate

**Figure 5:**
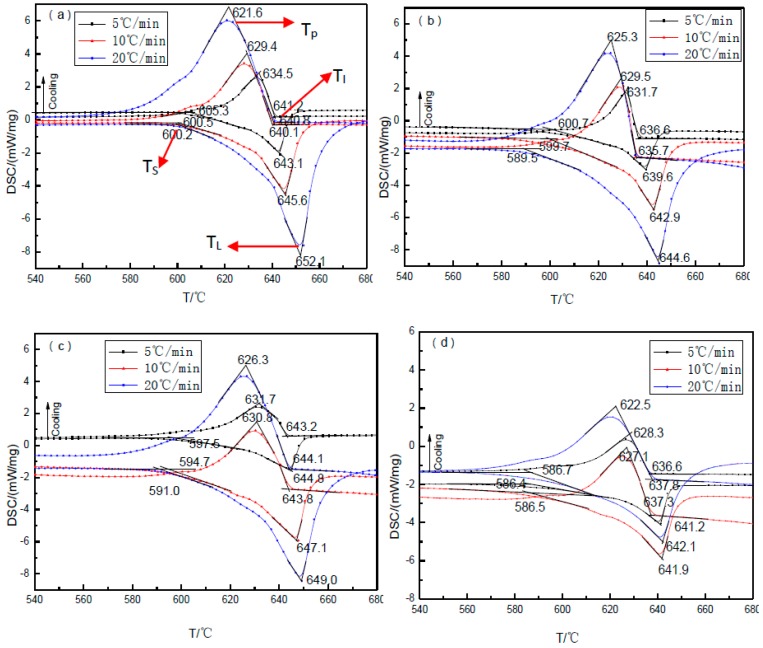
The differential scanning calorimetry (DSC) pattern of TiB_2_/7075 composites with different heating and cooling rates between 400 °C and 700 °C. (**a**) 3.0% TiB_2_/7075; (**b**) 4.5% TiB_2_/7075; (**c**) 6.0% TiB_2_/7075; (**d**) 9.0% TiB_2_/7075.

The solidus temperatures of TiB_2_/7075 Al matrix composites decreased with the increase of heating rate; the liquidus temperatures of TiB_2_/7075 Al matrix composites were the opposite, but the solidus and liquidus temperatures of 9.0% TiB_2_/7075 Al matrix composites showed no remarkable change, as seen in Figure 5 and Table 1. T_L_ and T_s_ are the liquidus and solidus temperature, respectively; T_I_ and T_P_ are the initial solidification temperature and peak temperatures of solidification, respectively. The nucleation needs to satisfy the condition with $\Delta T>\Delta T_{N}$ [22],

(1) $$\Delta T_{N}=T_{L}-T_{I}$$

where $\Delta T_{N}$ is nucleation undercooling. The solidus temperatures of TiB_2_/7075 Al matrix composites are on the decline, but the liquidus temperatures of TiB_2_/7075 Al matrix composites first decrease, then increase, and finally decrease again with the increase of TiB_2_ content. The liquidus and solidus temperatures of 7075 Al alloy were 637.7 °C and 475.0 °C, respectively, with heating rate of 10 °C/min [24]. The gap between the liquidus and solidus temperatures was 162.7 °C, but the maximum and minimum temperature differences of TiB_2_/7075 Al matrix composites were 54.4 °C and 43.2 °C, respectively, which are only about a third and a quarter of 7075 Al alloy. Pysz et al. also found that the liquidus and solidus temperatures of 7075 Al alloy were 637.0 °C and 475.5 °C, respectively. The initial solidification temperature and peak temperatures of solidification were 632.8 °C and 625.1 °C [25]. The gap between the liquidus and solidus temperatures was 161.5 °C, and the nucleation undercooling of 7075 Al alloy was 4.2 °C.

The initial solidification temperatures of 3.0% and 4.5% TiB_2_/7075 Al matrix composites decrease with the increase of cooling rate, but 6.0% and 9.0% TiB_2_/7075 Al matrix composites are the opposite. The peak temperatures of solidification in TiB_2_/7075 composites all decreased with the increase of cooling rate. The initial solidification temperatures of TiB_2_/7075 Al matrix composites also firstly decrease, then increase, and finally decrease again with the increase of TiB_2_ content. The peak temperatures of solidification in TiB_2_/7075 Al matrix composites firstly increase, then decrease with the increase of TiB_2_ content at cooling rate of 10 and 20 °C/min, respectively, but keep decreasing with a cooling rate of 5 °C/min. The nucleation undercooling of TiB_2_/7075 Al matrix composites firstly increase, then decrease, and finally increase with the increase of TiB_2_ content at a cooling rate of 5 and 10 °C/min, respectively, but the nucleation undercooling of TiB_2_/7075 Al matrix composites keep decreasing at a cooling rate of 20 °C/min.

### 3.3. Effect of TiB_2_ Content on the Nucleation of 7075 Al Alloy

According to the free growth model [15,16], two basic parameters are the density of effective particle and the degree of nucleation undercooling, which have an effect on dendrites to equiaxed growth in refined alloys. It is assumed that the nucleation is so potent that the initial aluminum nucleus can easily form a thin coating on the surface of the TiB_2_ particles, and that the undercooling for free growth $\Delta T_{fg}$ is the critical factor for grain initiation. Quested [26] also found the undercooling for free growth $\Delta T_{fg}$ and the nucleant particle diameter ***d*** are simply related by

(2) $$\Delta T_{fg}=\frac{4\sigma }{\Delta S_{V}d}$$

where σ is the solid–liquid interfacial free energy, and $\Delta S_{V}$ is the entropy of fusion per unit volume. The grain sizes of TiB_2_/7075 composites firstly decrease, then increase, and finally decrease with the increase of TiB_2_ content from Figure 1, Figure 2, Figure 3 and Figure 4, existing inversely proportional to the nucleation undercooling at cooling rates of 5 and 10 °C/min, respectively, by Equation (2). It can be found that the nucleation undercooling of TiB_2_/7075 composites firstly increase, then decrease, and finally increase with the increase of TiB_2_ content at cooling rates 5 and 10 °C/min, respectively, from Table 1. However, it cannot truly reflect the effect of TiB_2_ content on the nucleation of 7075 Al alloy at large cooling rates such as 20 °C/min, so the nucleation undercooling of TiB_2_/7075 composites keep growing with the increase of TiB_2_ content.

The nucleation undercooling of 3.0% and 4.5%, 6.0% and 9.0% TiB_2_/7075 composites at a cooling rate of 5 °C/min are 1.9 °C and 3.0 °C, 1.6 °C and 4.6 °C, respectively. The nucleation undercooling of 3.0% and 4.5%, 6.0% and 9.0% TiB_2_/7075 composites at a cooling rate of 10 °C/min are 5.5 °C and 7.3 °C, 3.3 °C and 4.6 °C, respectively. The gap of nucleation undercooling in TiB_2_/7075 composites at cooling rates of 5 °C/min and 10 °C/min are 3.6 °C and 4.3 °C, 1.7 °C and 0 °C, respectively, with the increase of TiB_2_ content. The change of the nucleation undercooling reflects the change of the critical nucleation radius of particles. The large change of nucleation undercooling with cooling rate means a wide range in particle diameter; the small change means a narrow range of particle diameter. That is to say, the nucleation undercooling of 9.0% TiB_2_/7075 composites has almost no change with changing cooling rate, which means the range of particle diameters is relatively narrow.

**Figure 6:**
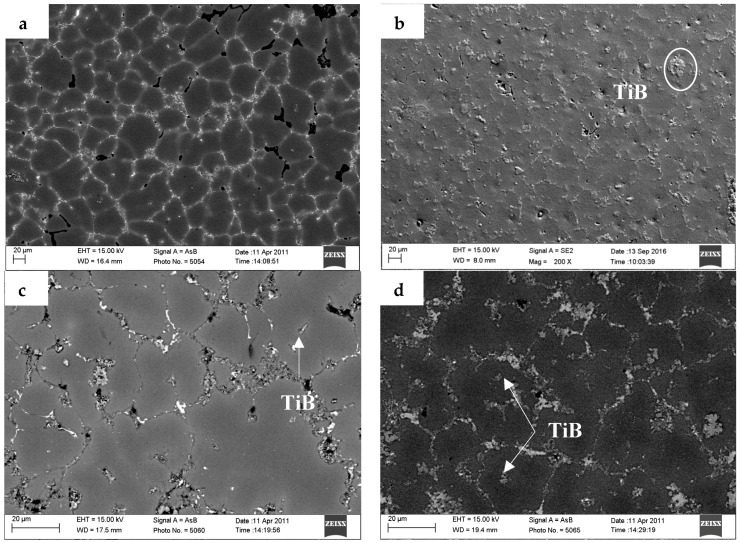
The microstructures of TiB_2_/7075 composites molten at 720 °C after being poured into the graphite mold. (**a**) 3.0% TiB_2_/7075; (**b**) 4.5% TiB_2_/7075; (**c**) 6.0% TiB_2_/7075; (**d**) 9.0% TiB_2_/7075.

**Figure 7:**
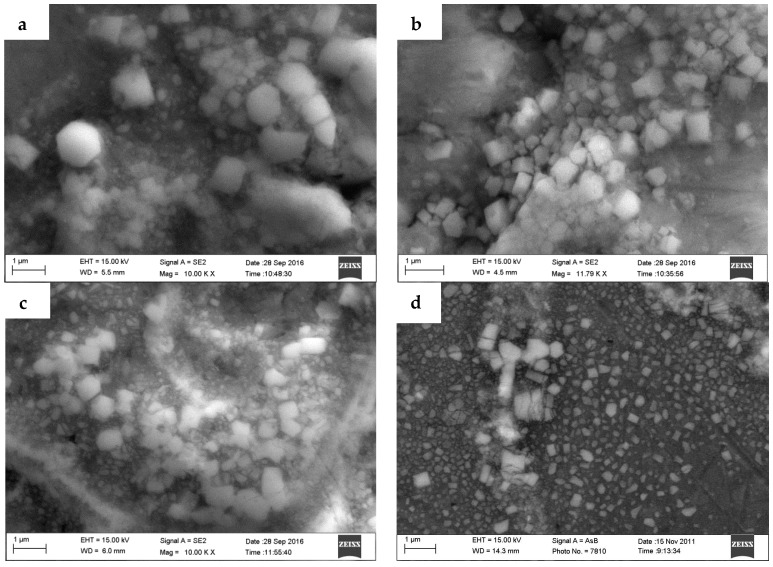
The micrograph of TiB_2_ particles in the composites. (**a**) 3.0% TiB_2_/7075; (**b**) 4.5% TiB_2_/7075; (**c**) 6.0% TiB_2_/7075; (**d**) 9.0% TiB_2_/7075.

Figure 6 shows the SEM images of as-cast TiB_2_/7075 composites. Most of the TiB_2_ particles appear to be located on α-Al grain boundaries; however, small agglomerates of TiB_2_ particles could be found in the grains of 6.0% and 9.0% TiB_2_/7075 composites. TiB_2_ particles are distributed in the grain boundary area; most particles were about 600–1000 nm in 3.0% and 4.5% TiB_2_/7075 composites in Figure 7a,b, and a minority of small TiB_2_ particles could also be found. The size of large TiB_2_ particles will decrease, the numbers of which will increase with increasing TiB_2_ particles, but the number of small TiB_2_ particles will also increase, because the heat of reaction of in-situ synthesis will also increase to refine the particles. More small TiB_2_ particles in 6.0% TiB_2_/7075 composites dispersed near large TiB_2_ particles in Figure 7c. Large TiB_2_ particles are distributed in the grain boundary, and a large number of scattered small TiB_2_ particles with size of about 400 nm were distributed in the grain near the grain boundary in Figure 7d. Decreasing of the size of large TiB_2_ particles will narrow the range of the size distribution; for example, the range of particle diameter changed from 1000–50 nm to 600–50 nm with the increase of TiB_2_ content, resulting in the change of nucleation undercooling.

**Figure 8:**
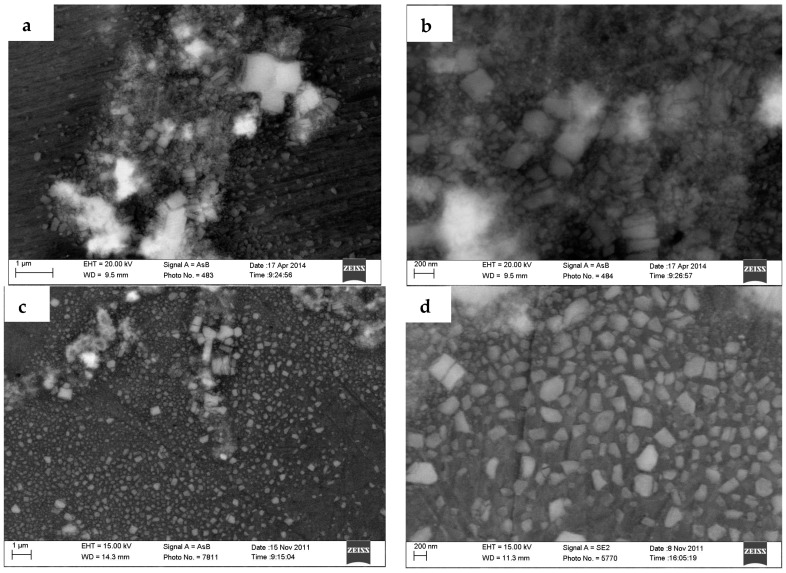

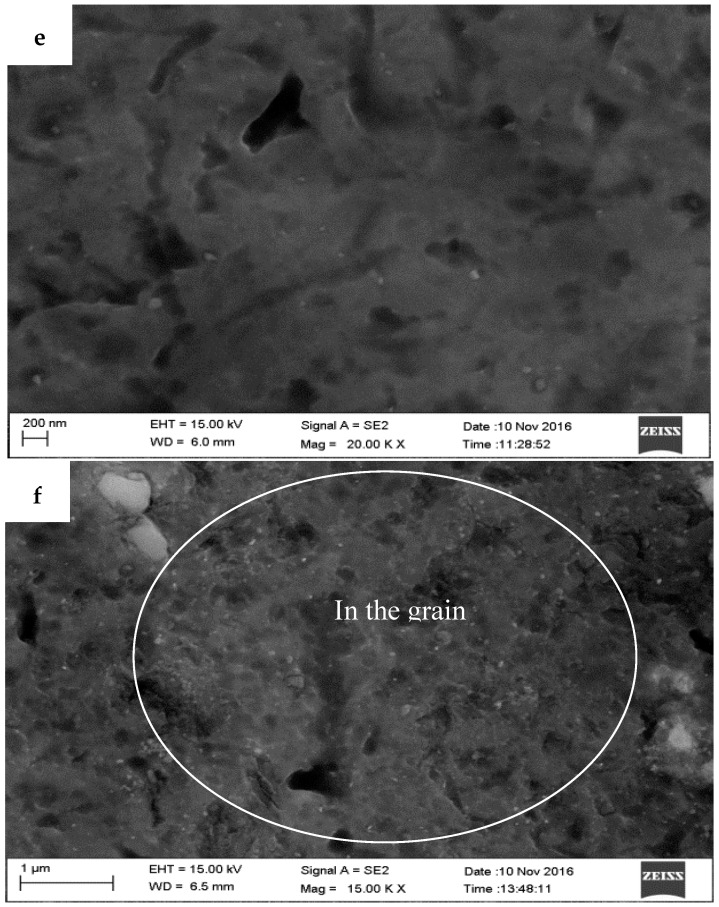
The distribution of TiB_2_ particles (**a**,**b**) in the grain boundary of 4.5% TiB_2_/7075; (**c**) in the grain and grain boundary of 9.0% TiB_2_/7075; (**d**) in the grain near the grain boundary of 9.0% TiB_2_/7075; (**e**) in the grain of 4.5% TiB_2_/7075; and (**f**) 9.0% TiB_2_/7075 (white dot in the picture).

The research has shown that the grain size varied from 73.58 μm to 12.75 μm, and then increased to 23.11 μm with the increase of reaction holding time [27]. At the same time, the mean size of TiB_2_ particles also varied from 570 nm to 430 nm, and then increased to 530 nm. Quested [20] also found the same tendencies—that the grain sizes of Al matrix composites firstly decreased and then grew again with decreasing of the average diameter of TiB_2_ particles, whereas the minimum grain size and the corresponding size of TiB_2_ particles were different in our research. TiB_2_ particles have the strongest nucleation abilities when the mean size of TiB_2_ particles is 250–500 nm, as shown in Figure 1 of [20]. TiB_2_ particles smaller than 600 nm can be found in the grain near the grain boundary in Figure 8a,c,d; most of the large TiB_2_ particles and parts of small TiB_2_ particles are distributed in the boundary area of grain in Figure 8b.

The nucleation at low cooling rates can best reflect the influence of particles on microstructures. We take the highest available value of σ = 158 mJ/m^2^ from contact-angle measurements, $\Delta S_{V}=1.112\times 10^{6}J/Km^{3}$ [16], it can be seen that the nucleant particle diameter of 4.5% and 9.0% TiB_2_/7075 Al matrix composites at a cooling rate of 5 °C/min is 188 nm and 123 nm, respectively, by Equation (2). From Figure 8e,f, more TiB_2_ particles are smaller than 200 nm (white dot in the picture) in the grain of 9.0% TiB_2_/7075 Al matrix composites, compared to 4.5% TiB_2_/7075 Al matrix composites. Comparing Figure 6, Figure 7 and Figure 8, we come to the conclusion that most particles smaller than 600 nm can act as heterogeneity nuclei, which will induce grain growth, and then TiB_2_ particles will be captured into the grain by solid/liquid interface. At the same time, most particles larger than 600 nm and a minority of small TiB_2_ particles are pushed into the grain boundary by the solid/liquid interface; locating in the grain boundary can hinder the Al atoms from diffusing during the solidification of the 7075 Al alloy and restrain α-Al phase growth. The mean size of TiB_2_ particles is relatively larger in 3.0% TiB_2_/7075 composites, which particles value a major role locating in the grain boundary. With increasing of TiB_2_ particles, the number of TiB_2_ particles smaller than 600 nm will increase, resulting in the increase of the capability of heterogeneous nucleus, and nucleation is the main function of TiB_2_ particles at contents below 4.5%. That is to say, the parts of TiB_2_ particle valve a major role locating in the grain boundary, and others become the nucleus of α-Al phase. The influence of particles shifted from dominating by locating to dominating by nucleation as the quantity of TiB_2_ increased, which causes a change from dendrites to uniform rosette grain, and again to large dendrites. Meanwhile, small agglomerates of TiB_2_ particles could also be found in TiB_2_/7075 composites with the increase of TiB_2_ content, because the tiny particles have a larger surface energy, even in 4.5% TiB_2_/7075 composites.

## 4. Conclusions

(1) The grain sizes of TiB_2_/7075 Al matrix composites firstly decreased, then increased, and finally decreased again with the increase of TiB_2_ content. The grain sizes of TiB_2_/7075 Al matrix composites increased with decreasing cooling rate, but the changes were not very noticeable from 20 °C/min to 5 °C/min. The mean grain size of 3.0% and 4.5% TiB_2_/7075 Al matrix composites could reach 236 μm and 165 μm, respectively, at a cooling rate of 1 °C/min.
(2) The liquidus temperatures of TiB_2_/7075 Al matrix composites firstly decreased, then increased, and finally decreased again with the increase of TiB_2_ content. The initial solidification temperatures and the nucleation undercooling of TiB_2_/7075 Al matrix composites firstly increased, then decreased, and finally increased again with the increase of TiB_2_ content. The melting and solidification process showed no significant change with the decrease of cooling rate in 9.0% TiB_2_/7075 Al matrix composites. A large change of the nucleation undercooling with cooling rate meant a wide range in particle diameter in TiB_2_/7075 composites, so a small change meant narrow range.
(3) Most small particles can act as heterogeneous nuclei, which induced the grain growth and were captured into the grain by solid/liquid interface. At the same time, most of larger particles and a minority of small TiB_2_ particles were pushed into the grain boundary, locating in the grain boundary can hinder the Al atoms from diffusing during the solidification of the 7075 Al alloy and restrain α-Al phase growth. The influence of particles shifted from dominating by locating to dominating by nucleation as the quantity of TiB_2_ particles increased due to the decrease of the average diameter of TiB_2_ particles.

## Figures and Tables

**Table 1:** Parameters of the melting and solidification process of composites.

Material	3% TiB_2_/7075			4.5% TiB_2_/7075			6% TiB_2_/7075			9% TiB_2_/7075		
Heating rate (°C·min^−1^)	5	10	20	5	10	20	5	10	20	5	10	20
Solidus temperature (°C)	605.3	600.5	600.2	600.7	599.7	589.5	597.5	594.7	591.0	586.4	586.5	586.7
Liquidus temperature(°C)	643.1	645.6	652.1	639.6	642.9	644.6	644.8	647.1	649.0	641.2	641.9	642.1
Temperature differences (°C)	37.8	44.9	51.9	38.9	43.2	55.1	47.3	52.4	58.0	54.8	54.4	55.6
Initial solidification temperature (°C)	641.2	640.1	640.8	636.6	635.6	635.6	643.2	643.8	644.1	636.6	637.3	637.8
Peak temperatures of solidification (°C)	634.5	629.4	621.6	631.7	629.5	625.3	631.7	630.8	626.3	628.3	627.1	622.5
Nucleation undercooling (°C)	1.9	5.5	11.4	3.0	7.3	9.0	1.6	3.3	4.9	4.6	4.6	4.3
